# Association Between Metabolic Syndrome and Its Components and the Risk of Head and Neck Cancer: A Systematic Review and Meta‐Analysis

**DOI:** 10.1002/cam4.71262

**Published:** 2025-09-26

**Authors:** Qingling Wang, Shiduo Guo, Ruizhe Huang, Zhenju Xu, Dapeng Liang, Yichuan Huang, Yubo Sun, Weiqi Yang, Liwei Jiang

**Affiliations:** ^1^ Department of Otolaryngology, Head and Neck Surgery The Affiliated Hospital of Qingdao University Qingdao Shandong China; ^2^ Department of Hepatobiliary Surgery Qingdao Jimo People's Hospital Qingdao Shandong China; ^3^ The First Clinical College Changsha Medical University Changsha China

**Keywords:** head and neck cancer, meta‐analysis, metabolic syndrome, relative risk, systematic review

## Abstract

**Background:**

The relationship between metabolic syndrome (MetS) and head and neck cancer (HNC) remains controversial. The present meta‐analysis evaluated the association between MetS and the risk of developing HNC.

**Methods:**

A literature search was conducted across various databases, including Embase, Cochrane, PubMed, and Web of Science, to identify studies investigating the relationship between MetS and the risk of HNC.

**Results:**

Twelve studies involving 55,692 participants were included. We found limited evidence of an association between MetS and the risk of developing HNC (RR = 1.1, 95% CI: 1.0–1.2, *p* = 0.07, I^2^ = 94%). Similar results were observed for HNC subtypes. Components of MetS revealed that underweight (BMI < 18.5 kg/m^2^) was associated with an increased risk of HNC (RR = 1.7, 95% CI: 1.5–1.9, *p* < 0.001). Low high‐density lipoprotein (HDL) cholesterol levels (RR = 1.0, 95% CI: 1.0–1.1, *p* = 0.003), hypertension (RR = 1.1, 95% CI: 1.0–1.1, *p* = 0.007), and diabetes (RR = 1.1, 95% CI: 1.0–1.2, *p* = 0.001) were associated with a minimal increase in the risk of HNC. However, high low‐density lipoproteins (LDL) cholesterol levels (RR = 0.8, 95% CI: 0.7–0.9, *p* < 0.001) and high total cholesterol levels (RR = 0.9, 95% CI: 0.9–0.9; *p* < 0.001) were associated with a reduced risk of HNC. Additionally, an increasing number of MetS components was associated with a higher risk of HNC.

**Conclusions:**

In conclusion, our meta‐analysis found little evidence of an association between MetS and the risk of developing HNC. However, high LDL and total cholesterol levels may be associated with a reduced risk of HNC, while being underweight may be associated with increased risk of HNC. These results need to be interpreted with caution due to the limited number of supporting studies.

## 1 Introduction

Head and neck cancer (HNC) is a heterogeneous group of malignancies affecting sites such as the oral cavity, oropharynx, hypopharynx, larynx, sinonasal cavity, and nasopharynx [1]. It is the seventh most commonly diagnosed cancer worldwide and poses a significant public health challenge [2]. The incidence of HNC has been steadily increasing, contributing to over 1.5% of cancer‐related deaths in the United States [3]. Given the high incidence and mortality rates of HNC, identifying and mitigating the risk factors and conditions associated with this cancer is imperative.

Metabolic syndrome (MetS) is a cluster of interconnected metabolic disorders, including hypertension, obesity, insulin resistance, dyslipidemia, and elevated triglycerides [4]. Numerous studies have established a connection between MetS and the risk of various malignancies, such as liver cancer [5], pancreatic cancer [6], colorectal cancer, and endometrial cancer [7, 8]. However, the relationship between MetS and HNC remains elusive, with previous studies reporting inconsistent results. While some studies found that MetS may heighten the risk of HNC and its subtypes, particularly oral and laryngeal cancers [9], others reported no significant association between MetS and the risk of HNC. However, strong correlations have been identified between the individual components of MetS and HNC risk [10]. Furthermore, some investigations propose that MetS may even reduce the risk of developing HNC [11].

To date, no comprehensive systematic review or meta‐analysis has specifically addressed the relationship between MetS and HNC. While one protocol has been published on this topic [12], it emphasizes the need for further investigation. Therefore, we conducted a meta‐analysis to evaluate the association between MetS and its components and the risk of HNC, to provide valuable insights that could enhance the current understanding and guide future research on this critical issue.

## 2 Methods

This research followed the guidelines outlined in the Meta‐analysis of Observational Studies in Epidemiology (MOOSE) Statement [13].

### 2.1 Search Strategy

A literature search was conducted across four databases, Embase, Cochrane, PubMed, and Web of Science, for all relevant English‐language literature published before October 31, 2024, utilizing primarily subject and free terms. The search parameters are detailed in Table S1.

### 2.2 Study Selection

The diagnostic criteria for MetS were based on the standards defined by the National Cholesterol Education Program Adult Treatment Panel III (NCEP‐ATP III), the American Heart Association (AHA), the International Diabetes Federation (IDF), and the China Diabetes Society (CDS). The inclusion criteria were based on the PICOS framework: (1) P (Participants): adult patients (aged ≥ 18 years); (2) I (Intervention): patients with MetS; (3) C (Control): patients without MetS; (4) O (Outcome): the primary outcomes were the incidence of HNC subtypes, including laryngeal cancer, oral cavity cancer, oropharyngeal cancer, hypopharyngeal cancer, nasopharyngeal cancer, nasal cavity cancer, and paranasal sinus cancer; (5) S (Study design): observational studies, including cohort studies (both prospective and retrospective) and case–control studies. The exclusion criteria were as follows: reviews, case reports, meta‐analyses, studies involving nonhuman populations, conference abstracts, commentaries, protocols, dissertations, editorials, studies that did not investigate MetS as the exposure, or studies with no relevant outcome.

### 2.3 Data Collection

Two authors collected the data included in the study using an Excel spreadsheet. The data collected included the first author, year of publication, country, cancer cases, MetS cases, location, study design, mean age or age range, study period, definition of MetS, risk of bias, and methodological quality in each study.

### 2.4 Assessment of Methodological Quality and Risk of Bias

The methodological quality of the included studies was evaluated by two authors using the Newcastle–Ottawa Scale (NOS), which assesses three main dimensions: selection, comparability, and outcome [14]. The scoring criteria were as follows: studies with scores of 0‐3 points were classified as low quality (high risk), those with 4‐6 points as moderate quality (medium risk), and those with 7‐9 points as high quality (low risk). Additionally, the Risk of Bias in Non‐Randomized Studies–of Interventions (ROBINS‐I) tool was utilized to evaluate the risk of bias in the studies. ROBINS‐I addresses critical factors, including selection bias, intervention bias, missing data, outcome assessment, confounding bias, and selective reporting. This tool systematically identifies potential biases to enhance the reliability of the results. Each study's risk of bias was categorized as low risk, moderate risk, or high risk. The rob_summary function in R was applied to generate a risk of bias chart. Disagreements in the assessments were resolved by the decision of a third independent reviewer.

### 2.5 Statistical Analysis

The indicators analyzed included the incidence of MetS, hazard ratios (HRs), odds ratios (ORs), relative risks (RRs), and 95% confidence intervals (CIs), all adjusted for multiple factors. For studies that reported only ORs and given the low incidence of HNC, we treated ORs as RRs. Similarly, for studies reporting only HRs, we treated HRs as RRs, assuming a roughly constant hazard over the follow‐up period. Since the event rates were low, these approximations introduced minimal bias, and no further conversions were performed. Heterogeneity between studies was assessed using *I* ^2^, with significant heterogeneity defined as *I* ^2^ > 50% and *p* < 0.1. In cases of significant heterogeneity, a random‐effects model was applied; otherwise, a fixed‐effects model was used. Sensitivity analyses were conducted to assess the impact of individual studies on the overall effect. Meta‐regression was employed to examine factors such as publication year, follow‐up time, study design, number of participants, MetS definition, geographical location, and HNC subtypes. Subgroup analyses were performed for significant variables. Publication bias was assessed using Egger's test, and all statistical tests were two‐tailed with a significance level of *p* < 0.05. Data were analyzed and visualized using R software version 4.3.3.

## 3 Results

### 3.1 Screening of Relevant Literature

**FIGURE 1:**
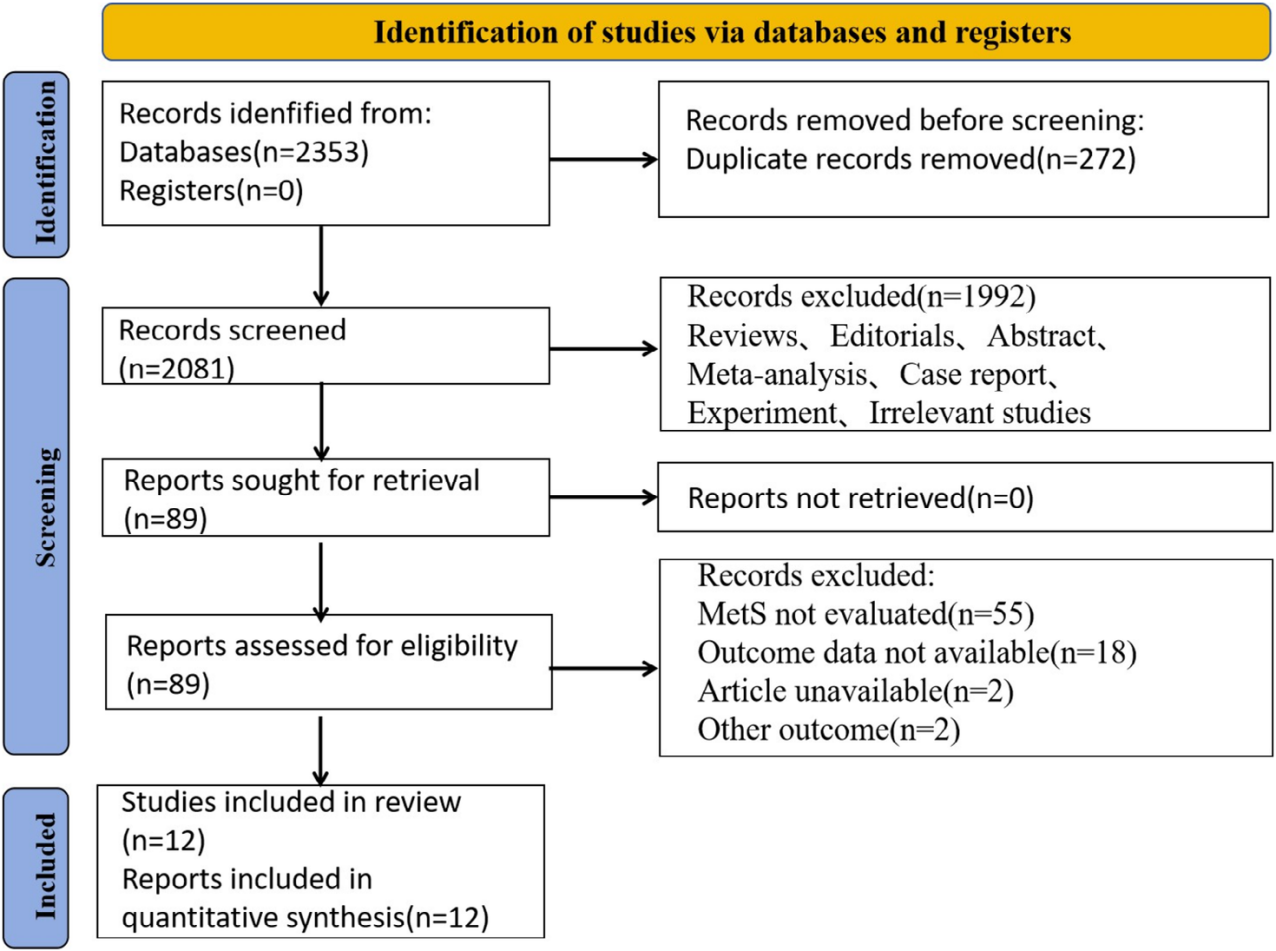
Flow chart of included studies.

A total of 2353 articles were retrieved from the Embase, Cochrane, PubMed, and Web of Science databases. After reviewing the titles and abstracts, 2264 duplicates and irrelevant articles were excluded, resulting in an initial selection of 89 articles. Following a full‐text review, 12 studies were included in the meta‐analysis, with one study addressing both oral and laryngeal cancer simultaneously (Figure 1).

### 3.2 Characteristics of Included Studies

The basic characteristics of the included studies are presented in Table 1. There were 10 cohort studies (9 retrospective cohort studies and 1 prospective cohort study) and 2 case–control studies. In total, 55,692 participants with HNC were analyzed, of whom 20,879 were diagnosed with MetS and 34,813 were not diagnosed with MetS. The overall age of the study cohort ranged from 18 to 99 years, and the studies were published between 2013 and 2023. The geographical distribution of the studies was as follows: nine studies from East Asia, two from Europe, and one from North America. For the definition of MetS, two studies employed the NEP‐ATP III criteria, eight referenced the IDF criteria, one used the CDS criteria, and one utilized the AHA criteria.

**TABLE 1:** Main characteristics of studies included in the meta‐analyses.

First author	Year of publication	Country	Cancer cases (*n*)	MetS cases (*n*)	Location	Study design	Mean age (year) or age range	Follow‐up time	Diagnostic criteria	Risk of bias	Quality	Adjustment confounders
Stott‐Mill [11]	2013	United States	14,022	3835	Oral cavity, oropharynx, larynx, hypopharynx	CC	68–99	1994–2007	NCEP‐ATP III	Moderate	7	Age, race, income, tobacco use, alcohol use, and registry
Zou [15]	2015	China	252	48	Oral cavity	RC	20–89	1998–2011	NCEP‐ATP III	High	7	Age, sex, pathologic grade, T category, N category, treatment
Zucchetto [16]	2018	Italy	197	17	Nasopharynx	CC	NA	1992–2008	IDF	Moderate	7	Age, sex, terms for study center, area of residence, period of interview, education, smoking and drinking habits
Kim [17]	2019	Korean	5322	2190	Larynx	RC	63.29 ± 9.71	2009–2015	IDF	Moderate	9	Age, sex, smoking status, alcohol intake, and exercise
Seo [18]	2020	Korean	6062	2240	Oral cavity	RC	NA	2009–2016	IDF	Moderate	9	Age, sex, smoking status, alcohol intake, exercise, and body mass index
Seo [18]	2020	Korean	2658	1049	Larynx	RC	NA	2009–2016	IDF	Moderate	9	Age, sex, smoking status, alcohol intake, exercise, and body mass index
Kim [19]	2021	Korean	1350	516	Larynx	RC	> 40	2009–2018	IDF	Moderate	9	Age, sex, smoking status, alcohol intake, exercise, and body mass index
Huang [20]	2021	China	2003	171	Nasopharynx	RC	18–78	2009–2012	CDS	High	8	Age, sex, smoking, WHO type and TNM stage
Jiang [21]	2021	UK	806	311	Oral cavity, oropharynx, larynx, hypopharynx, nasal cavity, paranasal sinus, nasopharynx	PC	37–73	2006–2015	AHA	Moderate	9	Age, sex, education, ethnicity, index of multiple deprivation, alcohol consumption, smoking status, physical activity, fruit and vegetable intake, NASIDS use, and CRP
Choi [22]	2022	Korean	8749	5540	Oral cavity	RC	58.52 ± 10.10	2009–2019	IDF	Moderate	9	Age, sex, smoking, drinking
Choi [10]	2022	Korean	2718	841	Oral cavity, oropharynx, larynx, hypopharynx, nasal cavity, paranasal sinus, nasopharynx	RC	59.53 ± 9.69	2008–2019	IDF	High	8	Age, sex
Kang [23]	2023	Korean	821	209	Hypopharynx	RC	61.54 ± 8.93	2008–2019	IDF	Moderate	8	Age, sex, smoking, and alcohol consumption
Kim [9]	2023	Korean	10,732	3912	Oral cavity, oropharynx, larynx, hypopharynx, nasal cavity, paranasal sinus, nasopharynx	RC	> 20	2009–2018	IDF	Moderate	9	Age, sex, smoking, alcohol consumption, regular exercise, income, diabetes, and hypertension

Abbreviations: AHA: American Heart, CC: case–control study, CDS: China Diabetes Society, IDF: International Diabetes Federation, n: number of samples, NCEP‐ATP III: National Cholesterol Education Program Adult Treatment Panel III, PC: prospective cohort study, RC: retrospective cohort study.

### 3.3 Assessment of Methodological Quality and Bias

**FIGURE 2:**
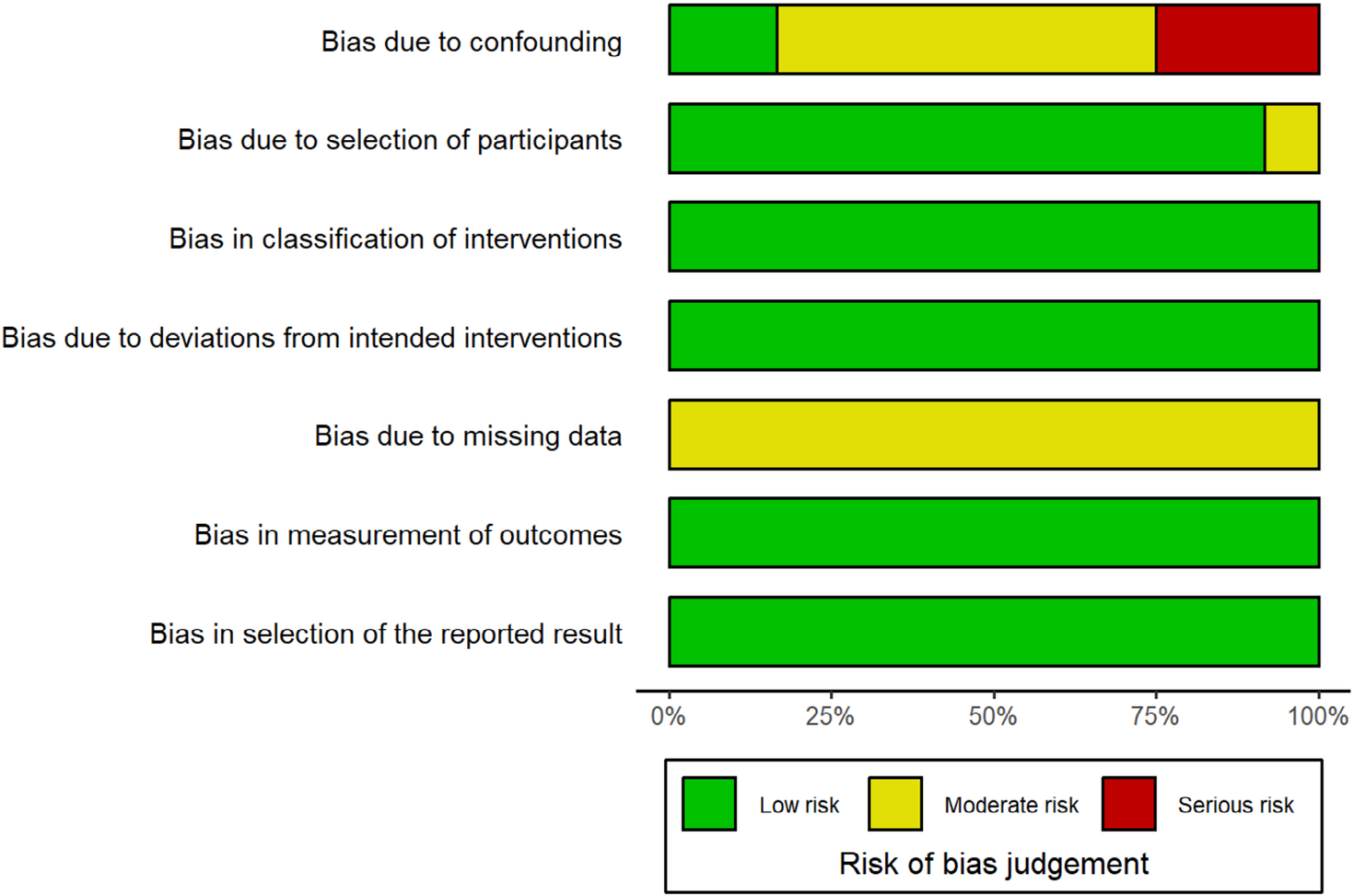
Summary risk of bias based on the ROBINS‐I tool.

The methodological quality assessment using the NOS scale showed a score of ≥ 7, classifying the study as high‐quality research (Table S2). The overall risk of bias across studies was assessed as follows: three studies were classified as high risk, while the others were classified as moderate risk. The high risk was primarily attributed to the failure to adjust for key confounders, such as chewing of betel nuts, smoking, and alcohol consumption (Table S3 and Figure 2). The results of Egger's test showed no evidence of publication bias; the *p* value for Egger's test was 0.71 (Figure S1).

### 3.4 Metabolic Syndrome in Association With Risk of HNC

**FIGURE 3:**
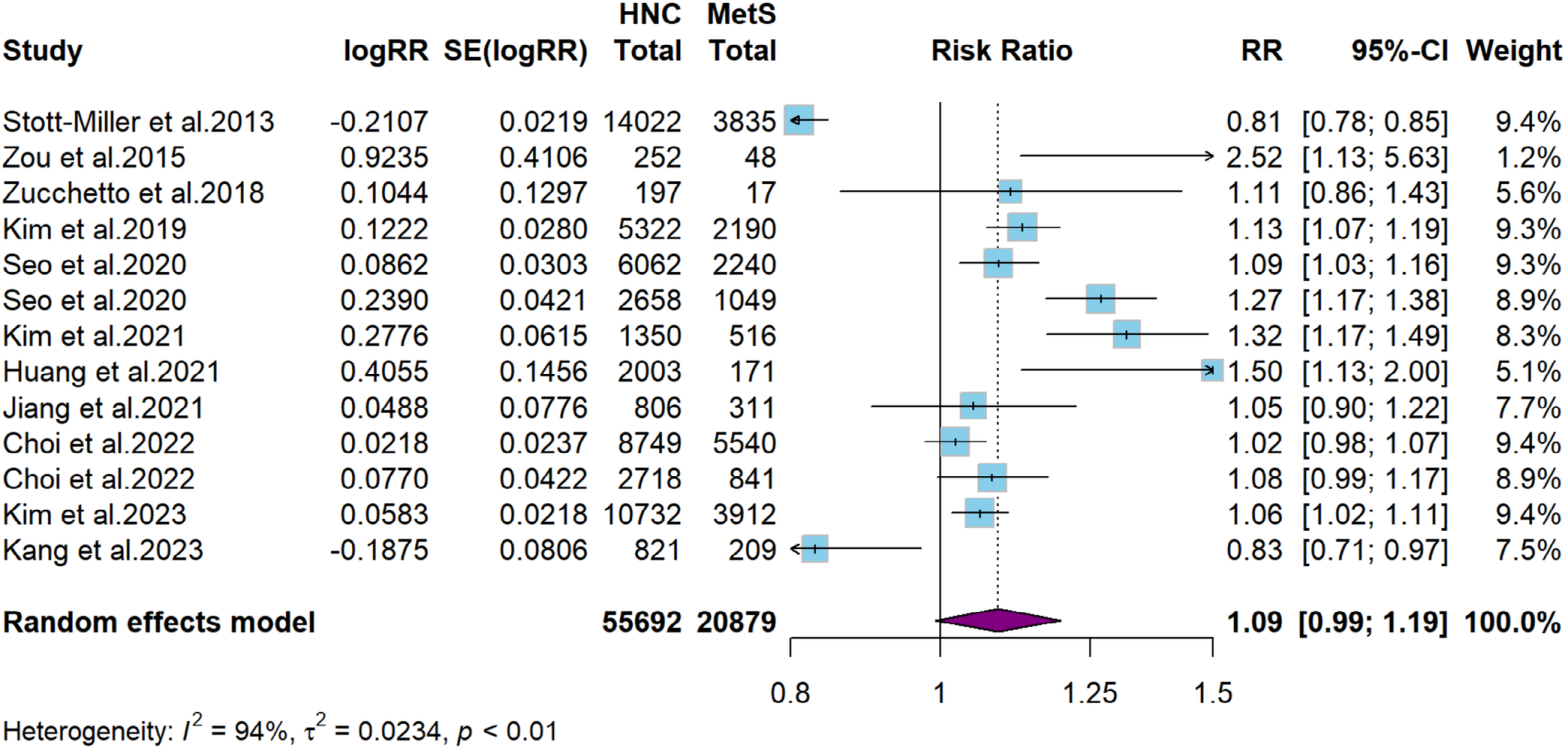
Meta‐analysis of MetS and risk of persons with HNC.

As shown in Figure 3, using a random‐effects model, the combined RR was 1.1 (95% CI: 1.0–1.2, *p* = 0.07). Significant heterogeneity between studies was observed (*I* ^2^ = 94%, *p* < 0.01). These findings suggested limited evidence of an association between MetS and the risk of developing HNC. Sensitivity analyses revealed that the RR of the total effect remained relatively stable, with an estimated range of 1.07‐1.12 after sequentially excluding each study, indicating the robustness of the overall results (Table 2 and Figure S2). The exclusion of the Stott–Miller study significantly reduced heterogeneity (*I* ^2^ = 80%, *p* = 0.004), although it remained high. To further explore the sources of heterogeneity, a meta‐regression analysis was conducted, examining potential factors such as the year of publication, study design, number of participants, geographic location, HNC subtype, follow‐up time, and MetS diagnostic criteria. The analysis revealed that study design (*p* = 0.04), geographic location (*p* = 0.02), and HNC subtype (*p* = 0.03) significantly contributed to heterogeneity (Table 3). In contrast, publication year, number of participants, follow‐up time, and diagnostic criteria had little effect on heterogeneity (*p* > 0.05). Subsequently, subgroup analyses of these factors were performed to further investigate their influence on the results.

**TABLE 2:** Sensitivity analysis.

Omitting study	RR (95% CI)	*p*	*I* ^2^
Omitting Stott‐Miller et al.	1.12 [1.04–1.20]	0.004	80%
Omitting Zou et al.	1.08 [0.98–1.18]	0.11	94%
Omitting Zucchetto et al.	1.09 [0.99–1.20]	0.09	94%
Omitting Kim et al.	1.09 [0.98–1.20]	0.11	94%
Omitting Seo et al.	1.09 [0.98–1.21]	0.10	94%
Omitting Seo et al.	1.07 [0.97–1.18]	0.16	94%
Omitting Kim et al.	1.07 [0.97–1.17]	0.16	94%
Omitting Huang et al.	1.07 [0.98–1.17]	0.15	94%
Omitting Jiang et al.	1.09 [0.99–1.21]	0.08	94%
Omitting Choi et al.	1.10 [0.99–1.22]	0.08	94%
Omitting Choi et al.	1.09 [0.981.21]	0.10	94%
Omitting Kim et al.	1.09 [0.99–1.21]	0.09	94%
Omitting Kang et al.	1.11 [1.01–1.22]	0.02	94%

**TABLE 3:** Meta‐regression analysis.

Covariate	*p*
Study design	0.04
Geographical location	0.02
HNC subtype	0.03
Year of publication	0.50
Number of participants	0.14
Follow‐up time	0.16
Diagnostic criteria	0.09

### 3.5 Analyses of Study Design and Geographic Location

**FIGURE 4:**
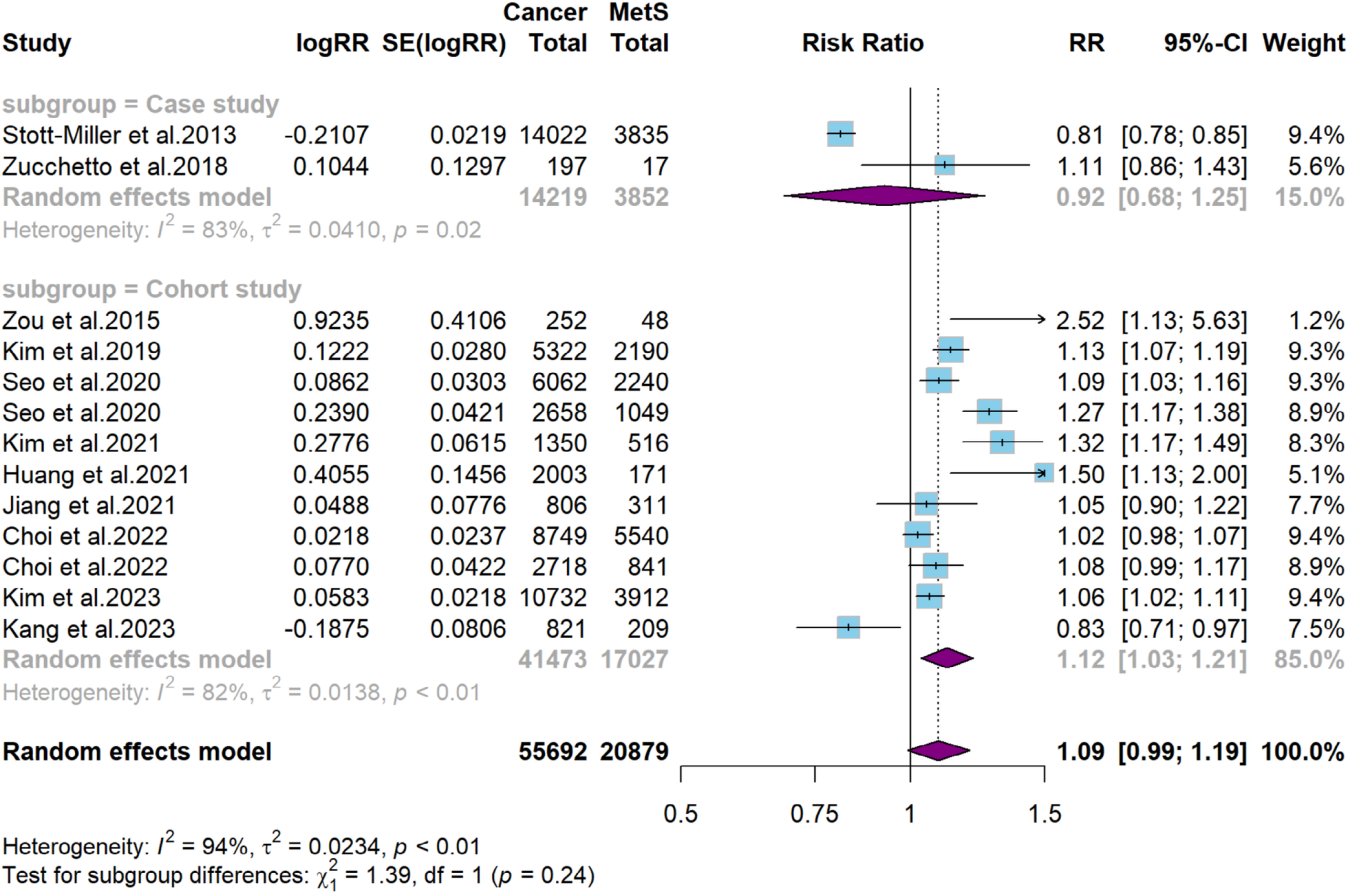
Subgroup analysis for the study design of MetS and risk of HNC.

**FIGURE 5:**
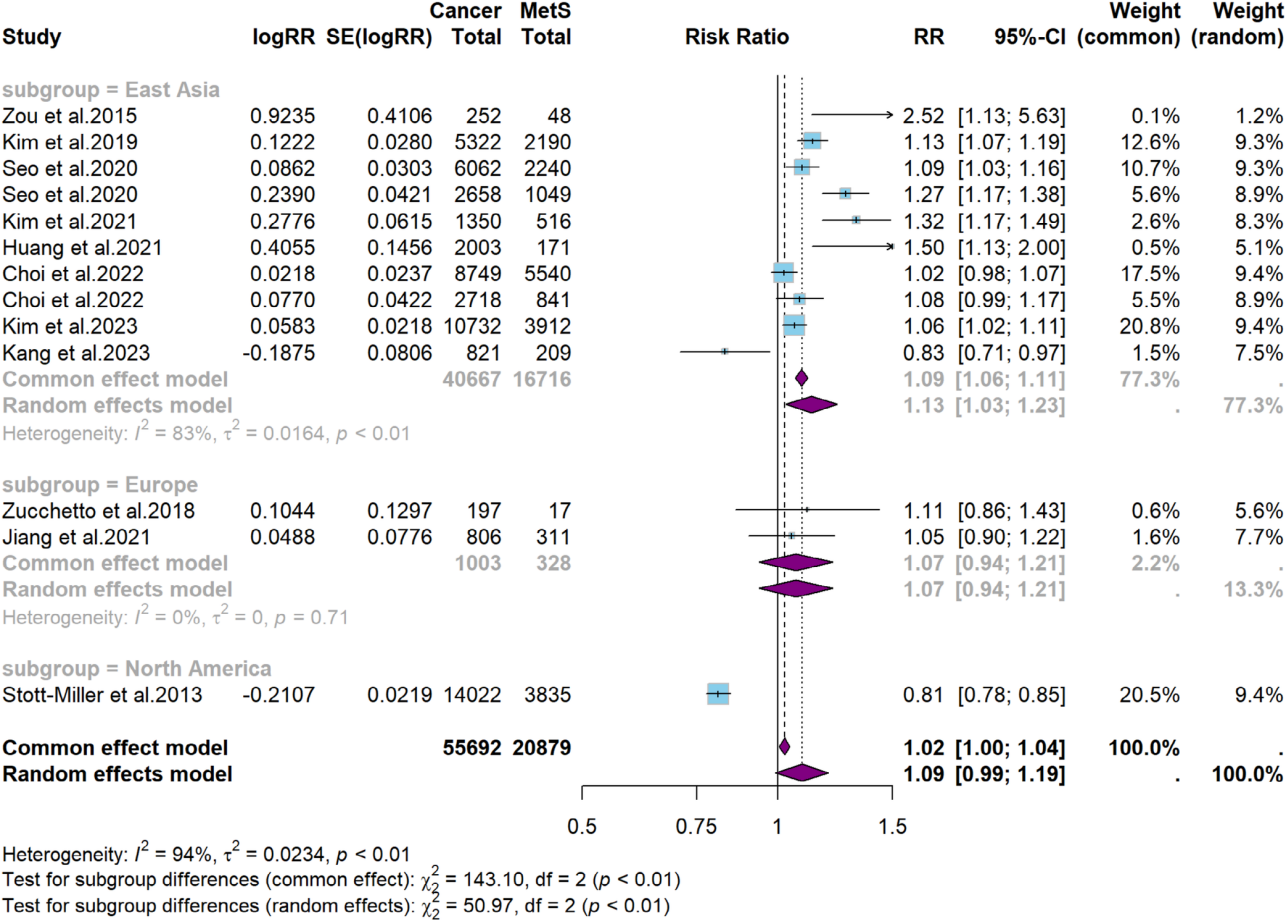
Subgroup analysis for the geographic location of MetS and risk of HNC.

In analyses stratified by study design, we found an association between MetS and an increased risk of HNC in cohort studies (RR = 1.1, 95% CI: 1.0–1.2, *p* = 0.01; *I* ^2^ = 82%), whereas case–control studies showed no such correlation (RR = 0.9, 95% CI: 0.7–1.3, *p >* 0.05, *I* ^2^ = 83%) (Figure 4). In analyses stratified by geographic location, we found an association between MetS and an increased risk of HNC in East Asia (RR = 1.1, 95% CI: 1.0–1.2, *p* = 0.01, *I* ^2^ = 83%). However, no significant association was found in Europe (RR = 1.1, 95% CI: 0.9–1.2, *p* = 0.32, *I* ^2^ = 0%). Remarkably, a study conducted in North America indicated that MetS was associated with a reduced risk of HNC (RR = 0.8, 95% CI: 0.8–0.9, *p* < 0.001) (Figure 5).

### 3.6 Subgroup Analyses of HNC Subtypes

**FIGURE 6:**
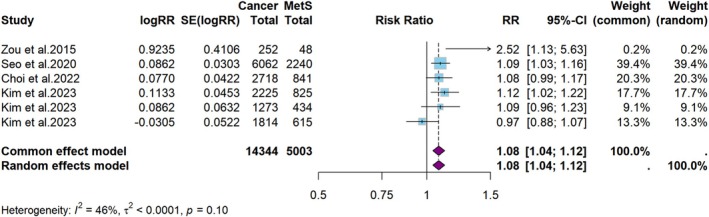
Meta‐analysis of the risk of oral cavity or oropharyngeal cancer in persons with MetS.

**FIGURE 7:**
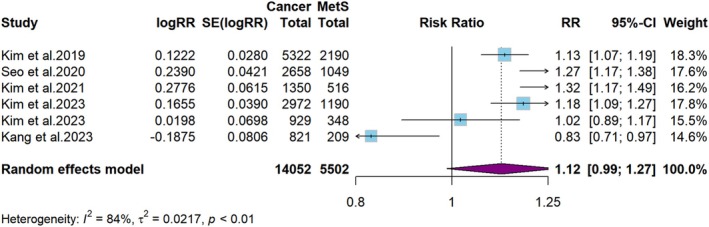
Meta‐analysis of the risk of laryngeal or hypopharyngeal cancer in persons with MetS.

**FIGURE 8:**
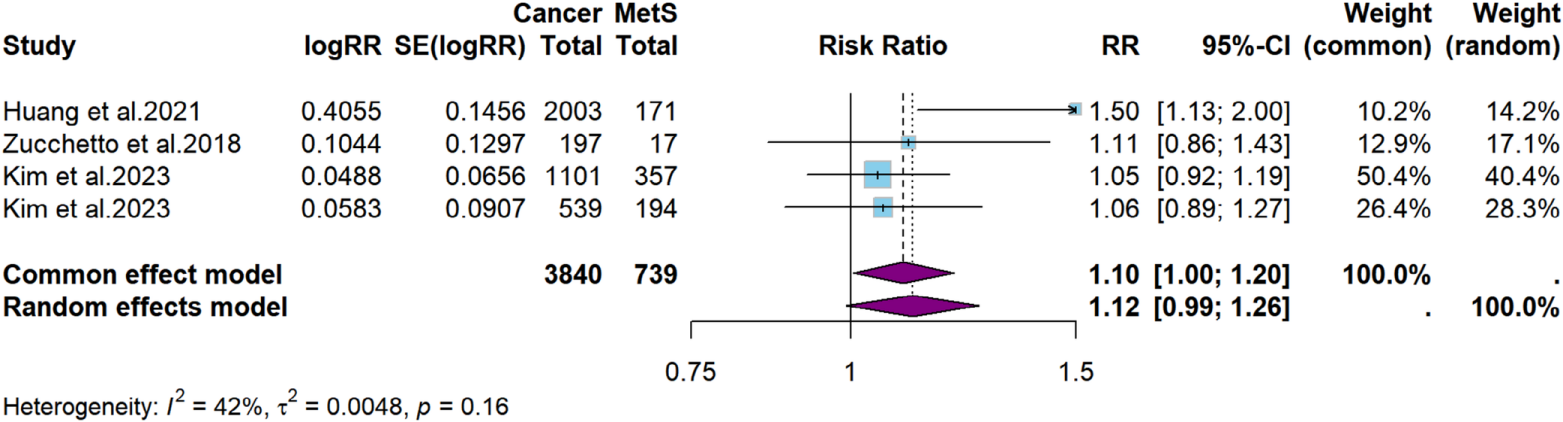
Meta‐analysis of the risk of sinonasal or nasopharyngeal cancer in persons with MetS.

The results of subgroup analyses examining the association between MetS and various subtypes of HNC are presented in Figures 6, 7, 8. The random‐effects model revealed that a minimal association exists between MetS and the risk of developing oral cavity or oropharyngeal cancers (RR = 1.1, 95% CI: 1.0–1.1, *p* < 0.001, *I* ^2^ = 46%, Figure 6). There was limited evidence suggesting that MetS was associated with an increased risk of laryngeal or hypopharyngeal cancers (RR = 1.1, 95% CI: 1.0–1.3, *p* = 0.07, *I* ^2^ = 84%, Figure 7), and sinonasal or nasopharyngeal cancers (RR = 1.1, 95% CI: 1.0–1.2, *p* = 0.07, *I* ^2^ = 42%, Figure 8).

### 3.7 Relationship Between the Number and Individual Components of MetS and Risk of HNC

The relationship between individual components of MetS and the risk of HNC is illustrated in Figure S3. Low high‐density lipoprotein (HDL) cholesterol levels (RR = 1.0, 95% CI: 1.0–1.1, *p* = 0.003), hypertension (RR = 1.1, 95% CI: 1.0–1.1, *p* = 0.007), and diabetes (RR = 1.1, 95% CI: 1.0–1.2, *p* = 0.001) were associated with a minimal increase in the risk of HNC. High low‐density lipoproteins (LDL) cholesterol levels (RR = 0.8, 95% CI: 0.7–0.9, *p* < 0.001) and high total cholesterol levels (RR = 0.9, 95% CI: 0.9–0.9; *p* < 0.001) were associated with a reduced risk of HNC.

**FIGURE 9:**
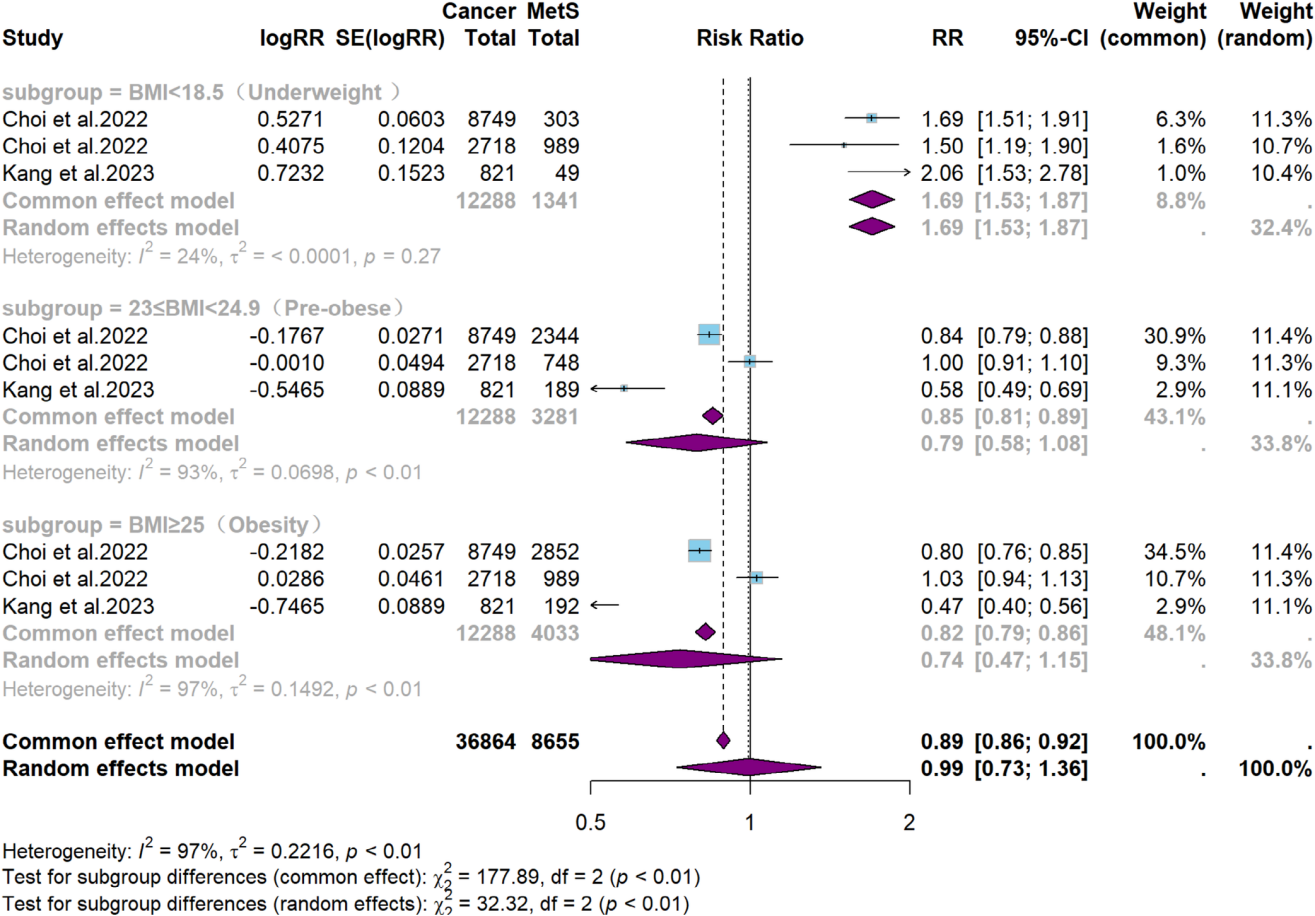
Meta‐analysis of the risk of HNC and BMI.

Body mass index (BMI) was categorized into four groups: underweight (< 18.5 kg/m^2^), normal weight (18.5–22.9 kg/m^2^), pre‐obese (23.0–24.9 kg/m^2^), and obesity (≥ 25 kg/m^2^). The correlation between BMI and HNC is depicted in Figure 9. Underweight was associated with an increased risk of HNC (RR = 1.7, 95% CI: 1.5–1.9, *p* < 0.001), whereas pre‐obese (RR = 0.8, 95% CI: 0.6–1.1, *p* = 0.14) and obesity were associated with a trend toward a lower risk of HNC (RR = 0.7, 95% CI: 0.5–1.2, *p* = 0.17), although the results were not statistically significant.

**FIGURE 10:**
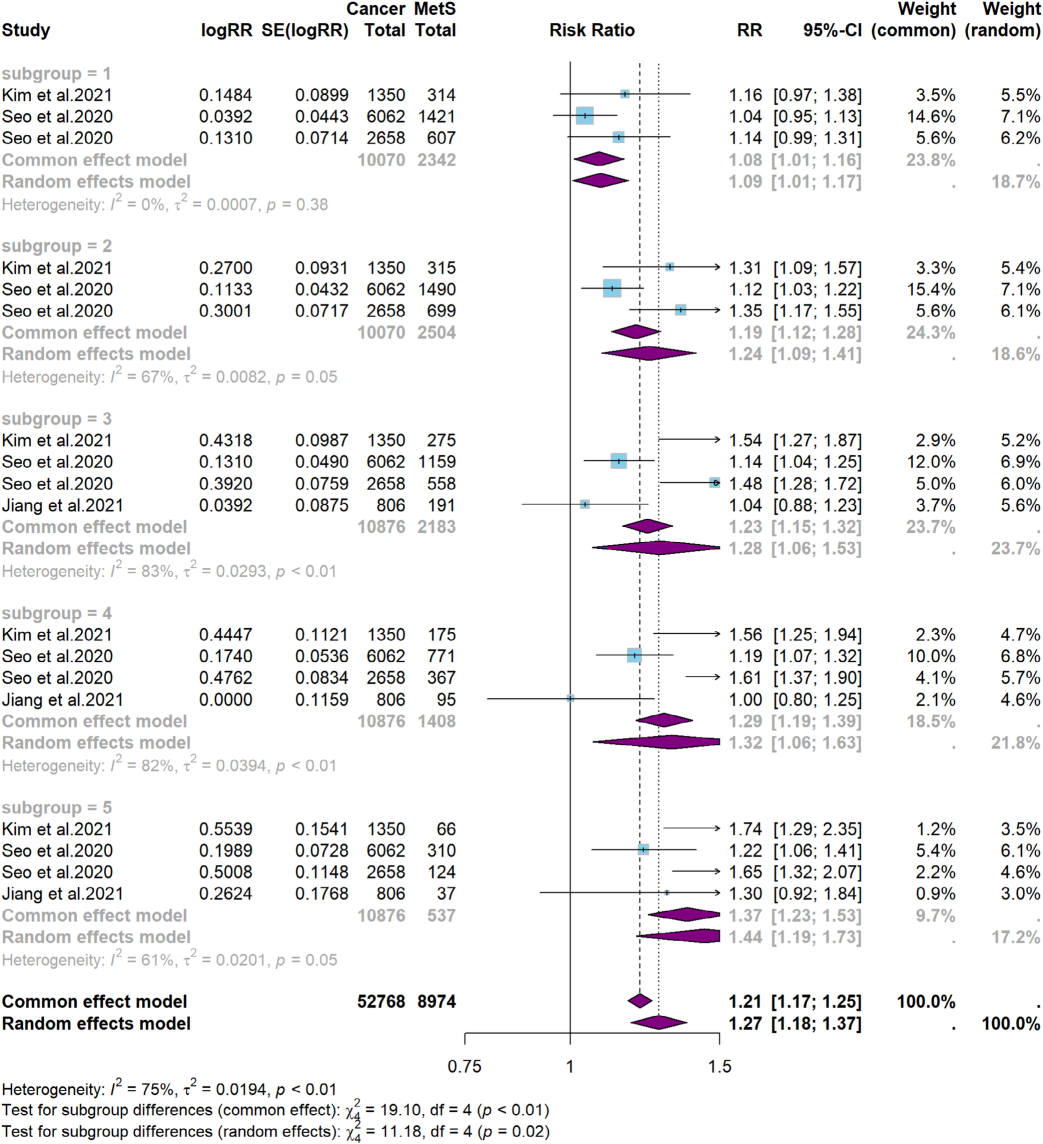
Meta‐analysis of the risk of HNC and the number of MetS components.

The relationship between the number of MetS components and the risk of HNC is displayed in Figure 10. It was observed that the risk of HNC was associated with an increase in the number of MetS components.

## 4 Discussion

This meta‐analysis found limited evidence of an association between MetS and the risk of developing HNC (RR = 1.1, 95% CI: 1.0–1.2, *p* = 0.07). Similar results were observed for HNC subtypes, particularly for laryngeal or hypopharyngeal cancers, and sinonasal or nasopharyngeal cancers. These results contrast with previous studies that reported conflicting findings: some studies found that MetS was associated with an increased risk of HNC [9], whereas others reported a protective effect [11]. Although our meta‐analysis found little evidence of an association between MetS and the risk of HNC, it does not rule out the potential biological or clinical relevance between the two conditions.

The observed substantial heterogeneity (*I* ^2^ = 94%) indicates that between‐study differences may significantly influence our findings, requiring careful consideration during interpretation. A sensitivity analysis was conducted to identify the source of this variability. The results demonstrated that part of the high heterogeneity may be attributed to the study by Stott‐Miller et al. [11], which found that MetS was associated with a reduced risk of HNC. The substantial discrepancy between the results of this study and the overall meta‐analysis may be because the study by Stott–Miller and colleagues focused on an American population and employed a nested case–control design, whereas most studies in our meta‐analysis were cohort studies conducted in East Asia. Additionally, meta‐regression analysis revealed that geographic location and study design contributed to the observed heterogeneity.

Subgroup analyses by geographical location revealed that MetS was associated with an increased risk of HNC in East Asia, whereas no significant association was found in Europe. This discrepancy suggests that factors such as lifestyle, environment, dietary habits, ethnicity, genetic predisposition, cultural background, and health management practices may play a crucial role in the relationship between MetS and HNC risk across populations in East Asia and Europe. In East Asia, habits such as betel chewing, high rates of smoking and alcohol consumption, and dietary patterns (characterized by high‐calorie and fat intake) contribute to a MetS phenotype more commonly associated with insulin resistance and hypertension [8, 24]. The impact of these factors may differ in Europe due to variations in lifestyle and health behaviors. Regarding subgroup analyses by study design, cohort studies revealed that MetS was associated with an increased risk of HNC, whereas no such association was found in case–control studies. This difference may be attributable to the smaller number of studies (*n* = 2) included in the case–control subgroup. Although the overall findings found little evidence of an association between MetS and HNC risk, subgroup analyses suggested an increased risk in East Asian populations and specific cohorts. These findings highlight the need for further investigation into region‐specific effects of MetS on HNC risk.

Additionally, the pathogenesis of HNC involves complex biological pathways. Components of MetS, such as hyperglycemia, hypertension, dyslipidemia, and insulin resistance, may contribute to the development of HNC. These effects may develop through chronic, cumulative processes and may not be adequately captured in conventional meta‐analyses. Notably, our subgroup analysis revealed that an increasing number of MetS components was progressively associated with a higher risk of HNC.

This suggests that the cumulative effects of MetS components may be correlated with HNC risk, despite the overall findings showing no significant association.

Previous studies demonstrated that both hypertension and diabetes may be associated with an increased risk of HNC and reported that these conditions could be risk factors for HNC development [25, 26, 27]. These metabolic conditions are believed to potentially induce immune system dysfunction and create a tumorigenic environment through various mechanisms, including oxidative stress and chronic systemic inflammation [28]. Additionally, these conditions are frequently associated with metabolic abnormalities, such as insulin resistance and disrupted glucose metabolism, both of which have been linked to the advancement of several cancers, including HNC [29]. Our data demonstrated that hypertension and diabetes were associated with a minimal increase in the risk of HNC. These findings emphasize the need for further investigation into the impact of individual MetS components and their cumulative effects on HNC to guide future clinical practice.

Currently, there is ongoing debate regarding the impact of obesity on the development of HNC. While some studies have found no direct association between obesity and the risk of HNC [30, 31], others suggest that obesity may contribute to an increased risk of these malignancies [32]. The present study found that underweight status was associated with an elevated risk of HNC, whereas pre‐obese or obesity showed a trend toward a reduced risk, although this association did not reach statistical significance. This finding is in line with previous studies that have reported a potential protective effect of a higher BMI against HNC and an increased likelihood of underweight individuals experiencing worse outcomes [33, 34]. Our findings reinforce the idea that both obesity and malnutrition influence HNC pathogenesis. Obesity has been linked to systemic changes, such as altered adipokine profiles, chronic low‐grade inflammation, and immune system dysregulation, all of which may create a tumor‐suppressive microenvironment, potentially reducing the likelihood of malignant transformation [35]. Conversely, malnutrition impairs immune function, tissue repair, and metabolic processes, resulting in immune suppression and heightened susceptibility to cancer. This highlights the critical role of nutritional status in influencing cancer risk [35].

LDL, HDL, and the LDL/HDL ratio serve as crucial prognostic indicators for the outcomes of HNC and its various subtypes [36]. Mechanistically, HDL plays an important role in regulating lipid balance and facilitating the removal of excess cholesterol, thereby supporting optimal tissue function [37]. In addition to its classic function in cholesterol transport, HDL particles exhibit multiple tumor‐suppressive effects. Furthermore, HDL may help reduce LDL accumulation in the vascular walls by attenuating oxidative cascades, which can lead to endothelial damage and atherosclerosis [38]. The protective role of HDL in cancer is well established. Our meta‐analysis found that low HDL levels were associated with a minimal increase in the risk of HNC. In contrast, the role of LDL and total cholesterol levels in tumor progression remains controversial. Previous studies found that compared with lower LDL levels, higher LDL levels were associated with poorer prognosis and shorter overall survival in HNC patients [39, 40]. Meanwhile, a recent study found that high total cholesterol levels were associated with an increased risk of HNC [41]. However, our meta‐analysis found that high LDL and total cholesterol levels were associated with a reduced risk of HNC. This observation aligns with the conclusions of Wilms et al. [36], whose research suggested that higher LDL levels may improve survival rates in patients with squamous cell carcinoma of the head and neck. Although our results found an association between total cholesterol levels and a reduced risk of HNC, this relationship may primarily be driven by LDL levels.

The association between high LDL levels and reduced HNC risk is unexpected, as it contradicts the general consensus that high LDL is a risk factor for both cardiovascular disease and cancer progression [42]. However, this conclusion should be interpreted with caution, particularly considering the potential reverse causality. For instance, in patients with HNC, LDL levels may decline in the early stages or during treatment due to weight loss and malnutrition. This does not imply a direct relationship between LDL levels and cancer risk. Furthermore, our study could not fully exclude statin use, which may have introduced reverse selection bias and obscured the true relationship between LDL levels and cancer risk. Specifically, the observed association may reflect the pharmacological effects of statins [43], such as their regulation of autophagy and induction of ferroptosis [44], rather than a direct relationship between high LDL levels and reduced risk of HNC. Moreover, insufficient adjustment for residual confounding factors may lead to overestimation or misinterpretation of the role of LDL levels in HNC risk.

Our meta‐analysis has several notable strengths. First, to the best of our knowledge, this is the first study to explore the relationship between the number and individual components of MetS and the risk of HNC and its subtypes, thereby filling a significant gap in the existing literature. Second, the methodological quality assessment conducted in our meta‐analysis indicated that the study is of high quality, thus enhancing the reliability and validity of our findings. Third, the sensitivity analysis results were consistent and stable, further validating the robustness of our conclusions. Finally, no significant publication bias was identified, ensuring the objectivity and comprehensiveness of the results.

Nevertheless, this meta‐analysis has several limitations. First, this substantial heterogeneity may limit the generalizability of the findings, despite being addressed through meta‐regression and subgroup analysis. Specifically, the high proportion of East Asian studies could introduce bias, as they may reflect region‐specific metabolic or lifestyle patterns that are not generalizable to other populations. Second, the included studies varied in their definitions of the diagnostic criteria for MetS, and some lacked data on central obesity or abdominal circumference, instead using BMI as a substitute. At the same time, we acknowledge that variations in the diagnostic criteria for MetS across different regions and sexes may impact the study results. Due to insufficient data, we were unable to explore these variables in this analysis. Third, this study primarily relies on observational studies (e.g., cohort or case–control studies), which are vulnerable to residual confounding that can never fully be adjusted for. As a result, there are significant limitations in establishing causal or temporal relationships between MetS and the development of HNC. Mendelian randomization is an effective method for causal inference and could help address some of these limitations. However, the absence of genetic data and insufficient sample size make it difficult to fully capture the complexity of MetS, limiting the applicability and integration of this approach in the current study. Fourth, excluding gray literature (e.g., master's/PhD theses, preprints) may have resulted in missing data. However, prioritizing peer‐reviewed sources emphasized rigor over potential publication bias, which could limit this meta‐analysis's comprehensiveness. Fifth, this study lacks registration in a protocol repository such as PROSPERO. Registration of systematic reviews and meta‐analyses is recommended to enhance transparency and reduce selective reporting, but this review was not registered before analysis. Future studies should register their protocols to improve rigor and transparency. Finally, only one prospective study design was included in this meta‐analysis. Future research should include more multicenter, multiethnic, prospective studies to validate our findings and further elucidate the mechanisms by which MetS influences HNC.

## 5 Conclusions

In summary, this meta‐analysis found limited evidence of an association between MetS and the risk of developing HNC. Underweight was associated with an increased risk, while high LDL and total cholesterol levels appeared to be linked with a reduced risk of HNC, which contrasts with conventional expectations. The cumulative effects of MetS components may contribute to the development of HNC. However, the results should be interpreted with caution, as they may be influenced by confounding factors such as tobacco, alcohol, and betel nut use, which were not consistently controlled for in the included studies. Furthermore, most of the studies were conducted in East Asia, which may limit the generalizability of these findings to other populations. To better validate these results, future research should focus on multicenter, multiethnic prospective studies.

## Author Contributions

The study was conceptualized and designed by Q.W. and L.J., who also contributed to the interpretation of the results. Data collection and analysis were carried out by S.G., R.H., Z.X., D.L., Y.H., Y.S., and W.Y. The initial manuscript draft was written by Q.W., while all authors participated in revising and refining the manuscript. The final version of the manuscript was reviewed, approved, and endorsed by all authors, ensuring the accuracy and integrity of the work.

## Ethics Statement

Since this is a review study, ethical approval was not required.

## Conflicts of Interest

The authors declare no conflicts of interest.

## Supporting information


**Table S1:** Search strategies for head and neck cancer.
**Table S2:** Methodological quality assessment of the included studies by NOS.
**Table S3:** Risk of bias of the included studies by ROBINS‐I.
**Figure S1:**. Publication bias test.
**Figure S2:**. Sensitivity analysis.
**Figure S3:** Meta‐analysis of each component of MetS with the risk of head and neck cancer.

## Data Availability

The raw data and results of this study can be found in the article and its Supporting Information. For inquiries, contact the corresponding author.
