# Supplementary material for: Association Between Metabolic Syndrome and Its Components and the Risk of Head and Neck Cancer: A Systematic Review and Meta‐Analysis
Source: Cancer Med. 2025 Sep 26;14(19):e71262. doi: 10.1002/cam4.71262 (PMC12464883; doi:10.1002/cam4.71262)
Supplement: Supplementary file 1 — Table S1: Search strategies for head and neck cancer. Table S2: Methodological quality assessment of the included studies by NOS. Table S3: Risk of bias of the included studies by ROBINS‐I. Figure S1:. Publication bias test. Figure S2:. Sensitivity analysis. Figure S3: Meta‐analysis of each component of MetS with the risk of head and neck cancer. [file CAM4-14-e71262-s001.docx]

**Supplementary Table 1.** Search strategies for Head and Neck Cancer.

| **Database** | **Search** |
| --- | --- |
| PubMed | ((((((((((((((((((((((((((((((((((Head and Neck Neoplasms[MeSH])) OR (Neoplasms, Head and Neck [Title/Abstract])) OR (Head, Neck Neoplasms [Title/Abstract])) OR (Head and Neck Neoplasm [Title/Abstract])) OR (Cancer of Head and Neck [Title/Abstract])) OR (Head and Neck Cancer [Title/Abstract])) OR (Cancer of the Head and Neck [Title/Abstract])) OR (Upper Aerodigestive Tract Neoplasms [Title/Abstract])) OR (UADT Neoplasm [Title/Abstract])) OR (Neoplasm, UADT [Title/Abstract])) OR (Neoplasms, UADT [Title/Abstract])) OR (UADT Neoplasms [Title/Abstract])) OR (Neoplasms, Upper Aerodigestive Tract [Title/Abstract])) OR (Upper Aerodigestive Tract Neoplasm [Title/Abstract])) OR (Head Neoplasms [Title/Abstract])) OR (Neoplasms, Head [Title/Abstract])) OR (Head Neoplasm [Title/Abstract])) OR (Neck Neoplasms [Title/Abstract])) OR (Neck Neoplasm [Title/Abstract])) OR (Neoplasm, Neck [Title/Abstract])) OR (Cancer of Head [Title/Abstract])) OR (Head Cancers [Title/Abstract])) OR (Head Cancer [Title/Abstract])) OR (Cancer, Head [Title/Abstract])) OR (Cancers, Head [Title/Abstract])) OR (Cancer of the Head [Title/Abstract])) OR (Cancer of Neck [Title/Abstract])) OR (Neck Cancers [Title/Abstract])) OR (Neck Cancer [Title/Abstract])) OR (Cancer, Neck [Title/Abstract])) OR (Cancers, Neck [Title/Abstract])) OR (Cancer of the Neck [Title/Abstract])) OR ((((((((((((((((Laryngeal Neoplasms [MeSH])) OR Neoplasms, Laryngeal [Title/Abstract])) OR (Laryngeal Cancer [Title/Abstract])) OR (Laryngeal Neoplasm [Title/Abstract])) OR (Larynx Neoplasms [Title/Abstract])) OR (Larynx Neoplasm [Title/Abstract])) OR (Neoplasm, Larynx [Title/Abstract])) OR (Neoplasms, Larynx [Title/Abstract])) OR (Cancer of Larynx [Title/Abstract])) OR (Larynx Cancers [Title/Abstract])) OR (Larynx Cancer [Title/Abstract])) OR (Cancer, Larynx [Title/Abstract])) OR (Cancers, Larynx [Title/Abstract])) OR (Cancer of the Larynx [Title/Abstract])) OR (Vocal Cord Cancer [Title/Abstract])) OR (Vocal Chord Cancer [Title/Abstract])) OR (Cancer, Vocal Cord [Title/Abstract])) OR (Cancer, Vocal Fold [Title/Abstract])) OR (Cancer, Subglottic [Title/Abstract])) OR (Cancer, Supraglottic [Title/Abstract])) OR (Throat Cancer [Title/Abstract])) OR ((((((((((((((((((((((((Mouth Neoplasms [MeSH])) OR Oral Cavity Cancer [Title/Abstract])) OR (Mouth Neoplasm [Title/Abstract])) OR (Neoplasm, Mouth [Title/Abstract])) OR (Neoplasms, Oral [Title/Abstract])) OR (Neoplasm, Oral [Title/Abstract])) OR (Oral Neoplasm [Title/Abstract])) OR (Oral Neoplasms [Title/Abstract])) OR (Neoplasms, Mouth [Title/Abstract])) OR (Cancer of Mouth [Title/Abstract])) OR (Mouth Cancers [Title/Abstract])) OR (Oral Cancer [Title/Abstract])) OR (Cancer, Oral [Title/Abstract])) OR (Cancers, Oral [Title/Abstract])) OR (Oral Cancers [Title/Abstract])) OR (Cancer of the Mouth [Title/Abstract])) OR (Mouth Cancer [Title/Abstract])) OR (Cancer, Mouth [Title/Abstract])) OR (Cancers, Mouth [Title/Abstract])) OR (Lip Cancer [Title/Abstract])) OR (Gum Cancer [Title/Abstract])) OR (Palatal Cancer [Title/Abstract])) OR (Gingiva Cancer [Title/Abstract])) OR (Buccal Cancer [Title/Abstract])) OR (Tongue Cancer [Title/Abstract])) OR (Lingual Cancer [Title/Abstract])) OR (Glosso Cancer [Title/Abstract])) OR ((((((((((((((((((((((Oropharyngeal Neoplasms [MeSH])) OR Neoplasm, Oropharyngeal [Title/Abstract])) OR (Oropharyngeal Neoplasm [Title/Abstract])) OR (Neoplasms, Oropharyngeal [Title/Abstract])) OR (Oropharynx Neoplasms [Title/Abstract])) OR (Neoplasm, Oropharynx [Title/Abstract])) OR (Oropharynx Neoplasm [Title/Abstract])) OR (Cancer of Oropharynx [Title/Abstract])) OR (Oropharynx Cancer [Title/Abstract])) OR (Cancer, Oropharyngeal [Title/Abstract])) OR (Cancers, Oropharyngeal [Title/Abstract])) OR (Oropharynx Cancers [Title/Abstract])) OR (Cancer of the Oropharynx [Title/Abstract])) OR (Cancers, Oropharynx [Title/Abstract])) OR ((((((((((((((((((Hypopharyngeal Neoplasms [MeSH])) OR Hypopharyngeal Neoplasm [Title/Abstract])) OR (Hypopharyngeal Cancer [Title/Abstract])) OR (Neoplasm, Hypopharyngeal [Title/Abstract])) OR (Neoplasms, Hypopharyngeal [Title/Abstract])) OR (Cancer, Hypopharyngeal [Title/Abstract])) OR (Cancers, Hypopharyngeal [Title/Abstract])) OR (Hypopharyngeal Cancers [Title/Abstract])) OR (Cancer, Epiglottis [Title/Abstract])) OR (Cancer, Arytenoids [Title/Abstract])) OR (Cancer, Aryepiglottic [Title/Abstract])) OR (Epiglottis Cancer [Title/Abstract])) OR (Arytenoids Cancer [Title/Abstract])) OR (Throat Cancer [Title/Abstract])) OR  (((((((((((((((((((((((((((Nasopharyngeal Neoplasms [MeSH])) OR (Nasopharyngeal Neoplasm [Title/Abstract])) OR (Neoplasm, Nasopharyngeal [Title/Abstract])) OR (Neoplasms, Nasopharyngeal [Title/Abstract])) OR (Nasopharynx Neoplasms [Title/Abstract])) OR (Nasopharynx Neoplasm [Title/Abstract])) OR (Neoplasm, Nasopharynx [Title/Abstract])) OR (Neoplasms, Nasopharynx [Title/Abstract])) OR (Cancer of Nasopharynx [Title/Abstract])) OR (Nasopharynx Cancers [Title/Abstract])) OR (Nasopharyngeal Cancer [Title/Abstract])) OR (Cancer, Nasopharyngeal [Title/Abstract])) OR (Cancers, Nasopharyngeal [Title/Abstract])) OR (Nasopharyngeal Cancers [Title/Abstract])) OR (Nasopharynx Cancer [Title/Abstract])) OR (Cancer, Nasopharynx [Title/Abstract])) OR (Cancers, Nasopharynx [Title/Abstract])) OR (Cancer of the Nasopharynx [Title/Abstract])) OR (Carcinoma, Nasopharyngeal [Title/Abstract])) OR (Carcinomas, Nasopharyngeal [Title/Abstract])) OR (Nasopharyngeal Carcinomas [Title/Abstract])) OR (Carcinoma of the Nasal Cavity [Title/Abstract])) OR (Carcinoma, Nasal Cavity [Title/Abstract])) OR (Cancers, Nasal Cavity [Title/Abstract])) OR (Cancer, Nasal Cavity [Title/Abstract])) OR (Paranasal Sinus Neoplasms [Title/Abstract])) OR (Neoplasm, Paranasal Sinus [Title/Abstract])) OR (Paranasal Sinus Neoplasm [Title/Abstract])) OR (Neoplasms, Paranasal Sinus [Title/Abstract])) OR (Paranasal Sinus Cancer [Title/Abstract])) OR (Cancer, Paranasal Sinus [Title/Abstract])) OR (Cancers, Paranasal Sinus [Title/Abstract])) OR (Paranasal Sinus Cancers [Title/Abstract])) OR (Cancer of Paranasal Sinus [Title/Abstract])) AND (((((((((((((((((((((((((((Metabolic Syndrome[MeSH])) OR (Metabolic Syndromes [Title/Abstract])) OR (Syndrome, Metabolic [Title/Abstract])) OR (Syndromes, Metabolic [Title/Abstract])) OR (Metabolic Syndrome X [Title/Abstract])) OR (Insulin Resistance Syndrome X [Title/Abstract])) OR (Syndrome X, Metabolic [Title/Abstract])) OR (Syndrome X, Insulin Resistance [Title/Abstract])) OR (Metabolic X Syndrome [Title/Abstract])) OR (Syndrome, Metabolic X [Title/Abstract])) OR (X Syndrome, Metabolic [Title/Abstract])) OR (Dysmetabolic Syndrome X [Title/Abstract])) OR (Syndrome X, Dysmetabolic [Title/Abstract])) OR (Reaven Syndrome X [Title/Abstract])) OR (Syndrome X, Reaven [Title/Abstract])) OR (Metabolic Cardiovascular Syndrome [Title/Abstract])) OR (Cardiovascular Syndrome, Metabolic [Title/Abstract])) OR (Cardiovascular Syndromes, Metabolic [Title/Abstract])) OR (Syndrome, Metabolic Cardiovascular [Title/Abstract])) OR (Cardiometabolic Syndrome [Title/Abstract])) OR (Cardiometabolic Syndromes [Title/Abstract])) OR (Syndrome, Cardiometabolic [Title/Abstract])) OR (Syndromes, Cardiometabolic [Title/Abstract])) OR (Dysregulated Metabolism [Title/Abstract])) OR (Metabolic Disorders [Title/Abstract])) OR (Metabolic Risk [Title/Abstract])) |
| Embase | ('Head and Neck Neoplasms'/exp OR 'Neoplasms, Head and Neck':ti,ab OR 'Head, Neck Neoplasms':ti,ab OR 'Head and Neck Neoplasm':ti,ab OR 'Cancer of Head and Neck':ti,ab OR 'Head and Neck Cancer':ti,ab OR 'Cancer of the Head and Neck':ti,ab OR 'Upper Aerodigestive Tract Neoplasms':ti,ab OR 'UADT Neoplasm':ti,ab OR 'Neoplasm, UADT':ti,ab OR 'Neoplasms, UADT':ti,ab OR 'UADT Neoplasms':ti,ab OR 'Neoplasms, Upper Aerodigestive Tract':ti,ab OR 'Upper Aerodigestive Tract Neoplasm':ti,ab OR 'Neoplasms, Laryngeal':ti,ab OR 'Laryngeal Cancer':ti,ab OR 'Laryngeal Neoplasm':ti,ab OR 'Larynx Neoplasms':ti,ab OR 'Larynx Neoplasm':ti,ab OR 'Neoplasm, Larynx':ti,ab OR 'Neoplasms, Larynx':ti,ab OR 'Cancer of Larynx':ti,ab OR 'Larynx Cancers':ti,ab OR 'Cancer, Larynx':ti,ab OR 'Oral Cavity Cancer':ti,ab OR 'Mouth Neoplasm':ti,ab OR 'Neoplasm, Mouth':ti,ab OR 'Neoplasms, Oral':ti,ab OR 'Neoplasm, Oral':ti,ab OR 'Oral Neoplasm':ti,ab OR 'Oral Neoplasms':ti,ab OR 'Cancer of Mouth':ti,ab OR 'Mouth Cancers':ti,ab OR 'Oral Cancer':ti,ab OR 'Cancer, Oral':ti,ab OR 'Oropharyngeal Neoplasm':ti,ab OR 'Oropharyngeal Cancer':ti,ab OR 'Neoplasms, Oropharyngeal':ti,ab OR 'Oropharynx Neoplasms':ti,ab OR 'Neoplasm, Oropharynx':ti,ab OR 'Oropharynx Neoplasm':ti,ab OR 'Cancer of Oropharynx':ti,ab OR 'Oropharynx Cancer':ti,ab OR 'Cancer, Oropharyngeal':ti,ab OR 'Hypopharyngeal Neoplasm':ti,ab OR 'Hypopharyngeal Cancer':ti,ab OR 'Neoplasm, Hypopharyngeal':ti,ab OR 'Neoplasms, Hypopharyngeal':ti,ab OR 'Cancer, Hypopharyngeal' OR 'Nasopharyngeal Neoplasms'/exp OR 'Nasopharyngeal Neoplasm':ti,ab OR 'Neoplasm, Nasopharyngeal':ti,ab OR 'Neoplasms, Nasopharyngeal':ti,ab OR 'Nasopharynx Neoplasms':ti,ab OR 'Nasopharynx Neoplasm':ti,ab OR 'Neoplasm, Nasopharynx':ti,ab OR 'Neoplasms, Nasopharynx':ti,ab OR 'Cancer of Nasopharynx':ti,ab OR 'Nasopharynx Cancers':ti,ab OR 'Nasopharyngeal Cancer':ti,ab OR 'Cancer, Nasopharyngeal':ti,ab OR 'Cancers, Nasopharyngeal':ti,ab OR 'Nasopharyngeal Cancers':ti,ab OR 'Nasopharynx Cancer':ti,ab OR 'Cancer, Nasopharynx':ti,ab OR 'Cancers, Nasopharynx':ti,ab OR 'Cancer of the Nasopharynx':ti,ab OR 'Carcinoma, Nasopharyngeal':ti,ab OR 'Carcinomas, Nasopharyngeal':ti,ab OR 'Nasopharyngeal Carcinomas':ti,ab OR 'Carcinoma of the Nasal Cavity':ti,ab OR 'Carcinoma, Nasal Cavity':ti,ab OR 'Cancers, Nasal Cavity':ti,ab OR 'Cancer, Nasal Cavity':ti,ab OR 'Paranasal Sinus Neoplasms'/exp OR 'Neoplasm, Paranasal Sinus':ti,ab OR 'Paranasal Sinus Neoplasm':ti,ab OR 'Neoplasms, Paranasal Sinus':ti,ab OR 'Paranasal Sinus Cancer':ti,ab OR 'Cancer, Paranasal Sinus':ti,ab OR 'Cancers, Paranasal Sinus':ti,ab OR 'Paranasal Sinus Cancers':ti,ab OR 'Cancer of Paranasal Sinus':ti,ab) AND ('Metabolic Syndrome'/exp OR 'Metabolic Syndromes':ti,ab OR 'Syndrome, Metabolic':ti,ab OR 'Syndromes, Metabolic':ti,ab OR 'Metabolic Syndrome X':ti,ab OR 'Insulin Resistance Syndrome X':ti,ab OR 'Syndrome X, Metabolic':ti,ab OR 'Syndrome X, Insulin Resistance':ti,ab OR 'Metabolic X Syndrome':ti,ab OR 'Syndrome, Metabolic X':ti,ab OR 'X Syndrome, Metabolic':ti,ab OR 'Dysmetabolic Syndrome X':ti,ab OR 'Syndrome X, Dysmetabolic':ti,ab OR 'Reaven Syndrome X':ti,ab OR 'Syndrome X, Reaven':ti,ab OR 'Metabolic Cardiovascular Syndrome':ti,ab OR 'Cardiovascular Syndrome, Metabolic':ti,ab OR 'Cardiovascular Syndromes, Metabolic':ti,ab OR 'Syndrome, Metabolic Cardiovascular':ti,ab OR 'Cardiometabolic Syndrome':ti,ab OR 'Cardiometabolic Syndromes':ti,ab OR 'Syndrome, Cardiometabolic':ti,ab OR 'Syndromes, Cardiometabolic':ti,ab OR 'Dysregulated Metabolism':ti,ab OR 'Metabolic Disorders':ti,ab OR 'Metabolic Risk':ti,ab) |
| Web of Science | (TS=("Head and Neck Neoplasms" OR "Neoplasms, Head and Neck" OR "Head, Neck Neoplasms" OR "Head and Neck Neoplasm" OR "Cancer of Head and Neck" OR "Head and Neck Cancer" OR "Cancer of the Head and Neck" OR "Upper Aerodigestive Tract Neoplasms" OR "UADT Neoplasm" OR "Neoplasm, UADT" OR "Neoplasms, UADT" OR "UADT Neoplasms" OR "Neoplasms, Upper Aerodigestive Tract" OR "Upper Aerodigestive Tract Neoplasm" OR "Head Neoplasms" OR "Neoplasms, Head" OR "Head Neoplasm" OR "Neck Neoplasms" OR "Neck Neoplasm" OR "Neoplasm, Neck" OR "Cancer of Head" OR "Head Cancers" OR "Head Cancer" OR "Cancer, Head" OR "Cancers, Head" OR "Cancer of the Head" OR "Cancer of Neck" OR "Neck Cancers" OR "Neck Cancer" OR "Cancer, Neck" OR "Cancers, Neck" OR "Cancer of the Neck" OR "Neoplasms, Laryngeal" OR "Laryngeal Cancer" OR "Laryngeal Neoplasm" OR "Larynx Neoplasms" OR "Larynx Neoplasm" OR "Neoplasm, Larynx" OR "Neoplasms, Larynx" OR "Cancer of Larynx" OR "Larynx Cancers" OR "Larynx Cancer" OR "Cancer, Larynx" OR "Cancers, Larynx" OR "Cancer of the Larynx" OR "Vocal Cord Cancer" OR "Vocal Chord Cancer" OR "Cancer, Vocal Cord" OR "Cancer, Vocal Fold" OR "Cancer, Subglottic" OR "Cancer, Supraglottic" OR "Throat Cancer" OR "Oral Cavity Cancer" OR "Mouth Neoplasm" OR "Neoplasm, Mouth" OR "Neoplasms, Oral" OR "Neoplasm, Oral" OR "Oral Neoplasm" OR "Oral Neoplasms" OR "Neoplasms, Mouth" OR "Cancer of Mouth" OR "Mouth Cancers" OR "Oral Cancer" OR "Cancer, Oral" OR "Cancers, Oral" OR "Oral Cancers" OR "Cancer of the Mouth" OR "Mouth Cancer" OR "Cancer, Mouth" OR "Cancers, Mouth" OR "Lip Cancer" OR "Gums Cancer" OR "Palatal Cancer" OR "Gingiva Cancer" OR "Buccal Cancer" OR "Tongue Cancer" OR "Lingual Cancer" OR "Glosso Cancer" OR "Neoplasm, Oropharyngeal" OR "Oropharyngeal Neoplasm" OR "Neoplasms, Oropharyngeal" OR "Oropharynx Neoplasms" OR "Neoplasm, Oropharynx" OR "Oropharynx Neoplasm" OR "Cancer of Oropharynx" OR "Oropharynx Cancer" OR "Cancer, Oropharyngeal" OR "Cancers, Oropharyngeal" OR "Oropharynx Cancers" OR "Cancer of the Oropharynx" OR "Cancers, Oropharynx" OR "Hypopharyngeal Neoplasm" OR "Hypopharyngeal Cancer" OR "Neoplasm, Hypopharyngeal" OR "Neoplasms, Hypopharyngeal" OR "Cancer, Hypopharyngeal" OR "Cancers, Hypopharyngeal" OR "Hypopharyngeal Cancers" OR "Cancer, Epiglottis" OR "Cancer, Arytenoids" OR "Cancer, Aryepiglottic" OR "Throat Cancer" OR "Nasopharyngeal Neoplasms" OR "Nasopharyngeal Neoplasm" OR "Neoplasm, Nasopharyngeal" OR "Neoplasms, Nasopharyngeal" OR "Nasopharynx Neoplasms" OR "Nasopharynx Neoplasm" OR "Neoplasm, Nasopharynx" OR "Neoplasms, Nasopharynx" OR "Cancer of Nasopharynx" OR "Nasopharynx Cancers" OR "Nasopharyngeal Cancer" OR "Cancer, Nasopharyngeal" OR "Cancers, Nasopharyngeal" OR "Nasopharyngeal Cancers" OR "Nasopharynx Cancer" OR "Cancer, Nasopharynx" OR "Cancers, Nasopharynx" OR "Cancer of the Nasopharynx" OR "Carcinoma, Nasopharyngeal" OR "Carcinomas, Nasopharyngeal" OR "Nasopharyngeal Carcinomas" OR "Carcinoma of the Nasal Cavity" OR "Carcinoma, Nasal Cavity" OR "Cancers, Nasal Cavity" OR "Cancer, Nasal Cavity" OR "Paranasal Sinus Neoplasms" OR "Neoplasm, Paranasal Sinus" OR "Paranasal Sinus Neoplasm" OR "Neoplasms, Paranasal Sinus" OR "Paranasal Sinus Cancer" OR "Cancer, Paranasal Sinus" OR "Cancers, Paranasal Sinus" OR "Paranasal Sinus Cancers" OR "Cancer of Paranasal Sinus")) AND (TS=("Metabolic Syndrome" OR "Metabolic Syndromes" OR "Syndrome, Metabolic" OR "Syndromes, Metabolic" OR "Metabolic Syndrome X" OR "Insulin Resistance Syndrome X" OR "Syndrome X, Metabolic" OR "Syndrome X, Insulin Resistance" OR "Metabolic X Syndrome" OR "Syndrome, Metabolic X" OR "X Syndrome, Metabolic" OR "Dysmetabolic Syndrome X" OR "Syndrome X, Dysmetabolic" OR "Reaven Syndrome X" OR "Syndrome X, Reaven" OR "Metabolic Cardiovascular Syndrome" OR "Cardiovascular Syndrome, Metabolic" OR "Cardiovascular Syndromes, Metabolic" OR "Syndrome, Metabolic Cardiovascular" OR "Cardiometabolic Syndrome" OR "Cardiometabolic Syndromes" OR "Syndrome, Cardiometabolic" OR "Syndromes, Cardiometabolic" OR "Dysregulated Metabolism" OR "Metabolic Disorders" OR "Metabolic Risk")) |
| Cochrane | ("Head and Neck Neoplasms" OR "Neoplasms, Head and Neck" OR "Head, Neck Neoplasms" OR "Head and Neck Neoplasm" OR "Cancer of Head and Neck" OR "Head and Neck Cancer" OR "Cancer of the Head and Neck" OR "Upper Aerodigestive Tract Neoplasms" OR "UADT Neoplasm" OR "Neoplasm, UADT" OR "Neoplasms, UADT" OR "UADT Neoplasms" OR "Neoplasms, Upper Aerodigestive Tract" OR "Upper Aerodigestive Tract Neoplasm" OR "Neoplasms, Laryngeal" OR "Laryngeal Cancer" OR "Laryngeal Neoplasm" OR "Larynx Neoplasms" OR "Larynx Neoplasm" OR "Neoplasm, Larynx" OR "Neoplasms, Larynx" OR "Cancer of Larynx" OR "Larynx Cancers" OR "Cancer, Larynx" OR "Oral Cavity Cancer" OR "Mouth Neoplasm" OR "Neoplasm, Mouth" OR "Neoplasms, Oral" OR "Neoplasm, Oral" OR "Oral Neoplasm" OR "Oral Neoplasms" OR "Cancer of Mouth" OR "Mouth Cancers" OR "Oral Cancer" OR "Cancer, Oral" OR "Oropharyngeal Neoplasm" OR "Oropharyngeal Cancer" OR "Neoplasms, Oropharyngeal" OR "Oropharynx Neoplasms" OR "Neoplasm, Oropharynx" OR "Oropharynx Neoplasm" OR "Cancer of Oropharynx" OR "Oropharynx Cancer" OR "Cancer, Oropharyngeal" OR "Hypopharyngeal Neoplasm" OR "Hypopharyngeal Cancer" OR "Neoplasm, Hypopharyngeal" OR "Neoplasms, Hypopharyngeal" OR "Cancer, Hypopharyngeal" OR "Nasopharyngeal Neoplasms" OR "Nasopharyngeal Neoplasm" OR "Neoplasm, Nasopharyngeal" OR "Neoplasms, Nasopharyngeal" OR "Nasopharynx Neoplasms" OR "Nasopharynx Neoplasm" OR "Neoplasm, Nasopharynx" OR "Neoplasms, Nasopharynx" OR "Cancer of Nasopharynx" OR "Nasopharynx Cancers" OR "Nasopharyngeal Cancer" OR "Cancer, Nasopharyngeal" OR "Cancers, Nasopharyngeal" OR "Nasopharyngeal Cancers" OR "Nasopharynx Cancer" OR "Cancer, Nasopharynx" OR "Cancers, Nasopharynx" OR "Cancer of the Nasopharynx" OR "Carcinoma, Nasopharyngeal" OR "Carcinomas, Nasopharyngeal" OR "Nasopharyngeal Carcinomas" OR "Carcinoma of the Nasal Cavity" OR "Carcinoma, Nasal Cavity" OR "Cancers, Nasal Cavity" OR "Cancer, Nasal Cavity" OR "Paranasal Sinus Neoplasms" OR "Neoplasm, Paranasal Sinus" OR "Paranasal Sinus Neoplasm" OR "Neoplasms, Paranasal Sinus" OR "Paranasal Sinus Cancer" OR "Cancer, Paranasal Sinus" OR "Cancers, Paranasal Sinus" OR "Paranasal Sinus Cancers" OR "Cancer of Paranasal Sinus") AND ("Metabolic Syndrome" OR "Metabolic Syndromes" OR "Syndrome, Metabolic" OR "Syndromes, Metabolic" OR "Metabolic Syndrome X" OR "Insulin Resistance Syndrome X" OR "Syndrome X, Metabolic" OR "Syndrome X, Insulin Resistance" OR "Metabolic X Syndrome" OR "Syndrome, Metabolic X" OR "X Syndrome, Metabolic" OR "Dysmetabolic Syndrome X" OR "Syndrome X, Dysmetabolic" OR "Reaven Syndrome X" OR "Syndrome X, Reaven" OR "Metabolic Cardiovascular Syndrome" OR "Cardiovascular Syndrome, Metabolic" OR "Cardiovascular Syndromes, Metabolic" OR "Syndrome, Metabolic Cardiovascular" OR "Cardiometabolic Syndrome" OR "Cardiometabolic Syndromes" OR "Syndrome, Cardiometabolic" OR "Syndromes, Cardiometabolic" OR "Dysregulated Metabolism" OR "Metabolic Disorders" OR "Metabolic Risk") |

**Supplementary Table 2** Methodological quality assessment of the included studies by NOS.

|  | **Selection** | | | |  | **Comparability** | |  | **Outcome** | | |  | **Total** |
| --- | --- | --- | --- | --- | --- | --- | --- | --- | --- | --- | --- | --- | --- |
| Cohort study  (n=10) | Representativeness of exposed cohort | Selection of non-exposed cohort | Ascertainment of exposure | Presentation of outcome as start |  | Control for important factor | Controls for any additional factor |  | Assessment of outcome | Sufficient follow-up time (five years) | Appropriate follow-up |  | Score (0-9) |
| Zou,2015 | 1 | 0 | 1 | 1 |  | 1 | 0 |  | 1 | 1 | 1 |  | 7 |
| Kim,2019 | 1 | 1 | 1 | 1 |  | 1 | 1 |  | 1 | 1 | 1 |  | 9 |
| Seo,2020 | 1 | 1 | 1 | 1 |  | 1 | 1 |  | 1 | 1 | 1 |  | 9 |
| Kim,2021 | 1 | 1 | 1 | 1 |  | 1 | 1 |  | 1 | 1 | 1 |  | 9 |
| Huang,2021 | 1 | 0 | 1 | 1 |  | 1 | 1 |  | 1 | 1 | 1 |  | 8 |
| Jiang,2021 | 1 | 1 | 1 | 1 |  | 1 | 1 |  | 1 | 1 | 1 |  | 9 |
| Choi,2022 | 1 | 1 | 1 | 1 |  | 1 | 1 |  | 1 | 1 | 1 |  | 9 |
| Choi,2022 | 1 | 1 | 1 | 1 |  | 1 | 0 |  | 1 | 1 | 1 |  | 8 |
| Kang, 2023 | 1 | 1 | 1 | 1 |  | 1 | 0 |  | 1 | 1 | 1 |  | 8 |
| Kim,2023 | 1 | 1 | 1 | 1 |  | 1 | 1 |  | 1 | 1 | 1 |  | 9 |
| Case-control study  (n=2) | **Selection** | | | |  | **Comparability** |  |  | **Exposure** | | |  | **Total** |
|  | Adequate definition of cases | Representativeness of cases | Selection of control | Definition of control |  | Control for important factor | Controls for any additional factor |  | Ascertainment of exposure | Same method of ascertainment | Non-response rate |  | Score (0-9) |
| Stott-Miller,2013 | 1 | 1 | 1 | 1 |  | 1 | 0 |  | 1 | 1 | 0 |  | 7 |
| Zucchetto, 2018 | 1 | 1 | 1 | 1 |  | 1 | 0 |  | 1 | 1 | 0 |  | 7 |

**Supplementary Table 3** Risk Of Bias of the included studies by ROBINS-I.

| Study | Bias due to confounding | Bias due to selection of participants | Bias due to classification of interventions | Bias due to measurement of outcomes | Bias due to missing data | Bias in reporting of results | Other biases | Total bias |
| --- | --- | --- | --- | --- | --- | --- | --- | --- |
| Stott-Miller et al.2013 | Moderate | Moderate | Low | Low | Moderate | Low | Low | Moderate |
| Zou et al.2015 | High | Low | Low | Low | Moderate | Low | Low | High |
| Zucchetto et al.2018 | Moderate | Low | Low | Low | Moderate | Low | Low | Moderate |
| Kim et al.2019 | Moderate | Low | Low | Low | Moderate | Low | Low | Moderate |
| Seo et al.2020 | Moderate | Low | Low | Low | Moderate | Low | Low | Moderate |
| Kim et al.2021 | Moderate | Low | Low | Low | Moderate | Low | Low | Moderate |
| Huang et al.2021 | High | Low | Low | Low | Moderate | Low | Low | High |
| Jiang et al.2021 | Moderate | Low | Low | Low | Moderate | Low | Low | Moderate |
| Choi et al.2022 | Moderate | Low | Low | Low | Moderate | Low | Low | Moderate |
| Choi et al.2022 | High | Low | Low | Low | Moderate | Low | Low | High |
| Kang et al.2023 | Moderate | Low | Low | Low | Moderate | Low | Low | Moderate |
| Kim et al.2023 | Moderate | Moderate | Low | Low | Moderate | Low | Low | Moderate |

**
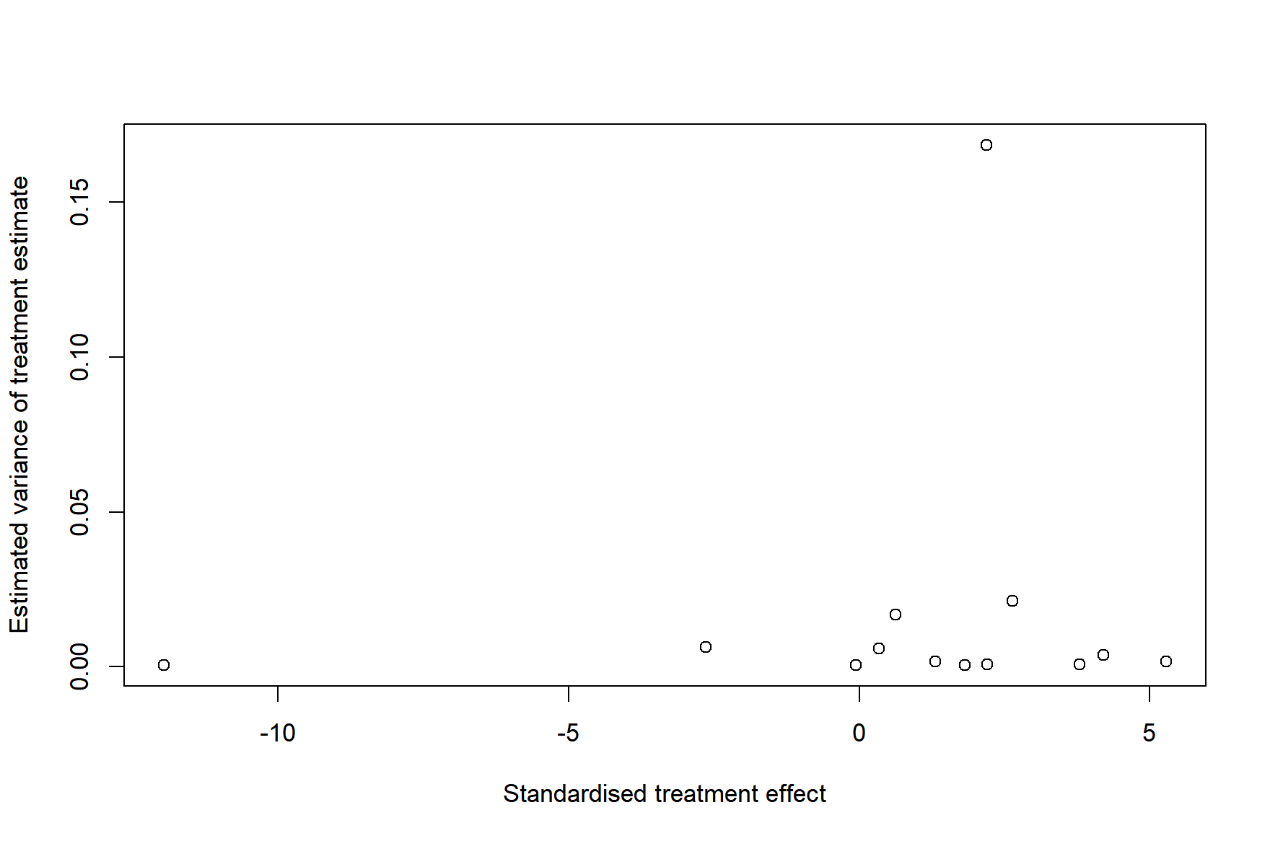
**

**Supplementary Figure 1.** **Publication bias test.**


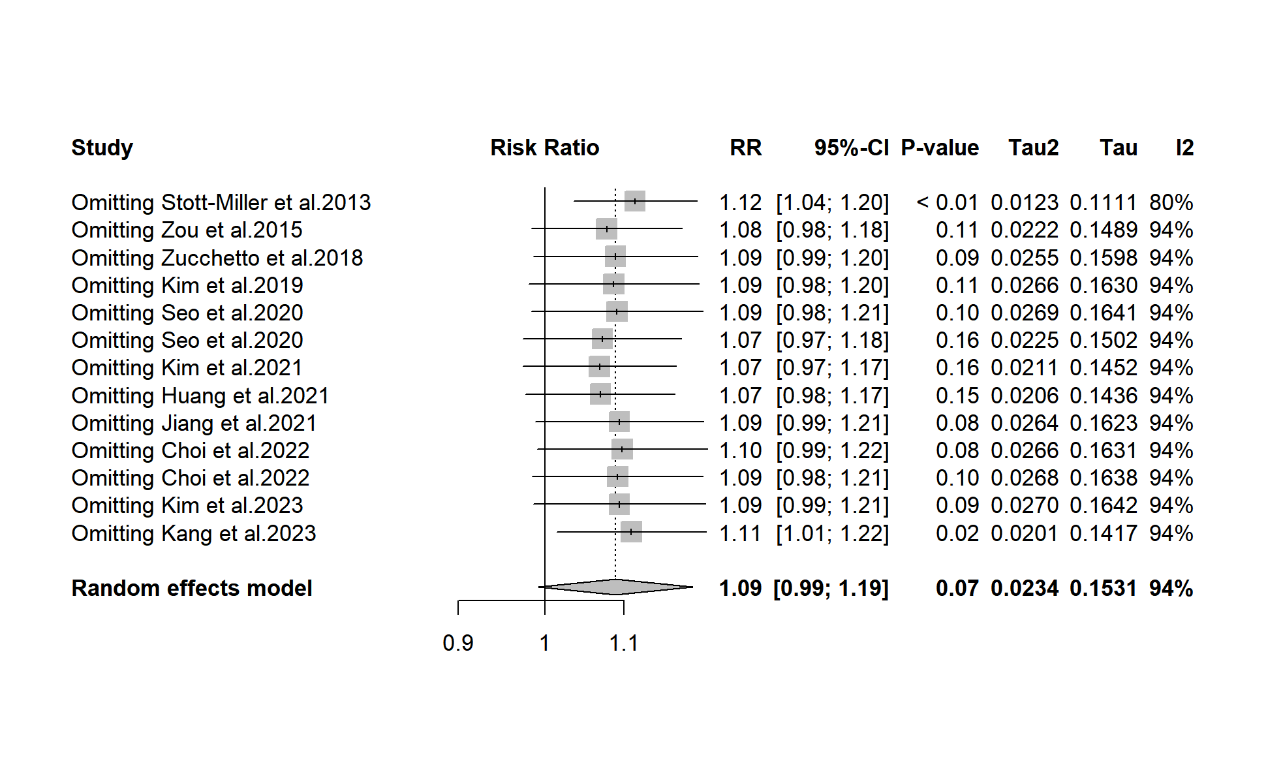


**Supplementary Figure 2.** **Sensitivity analysis**


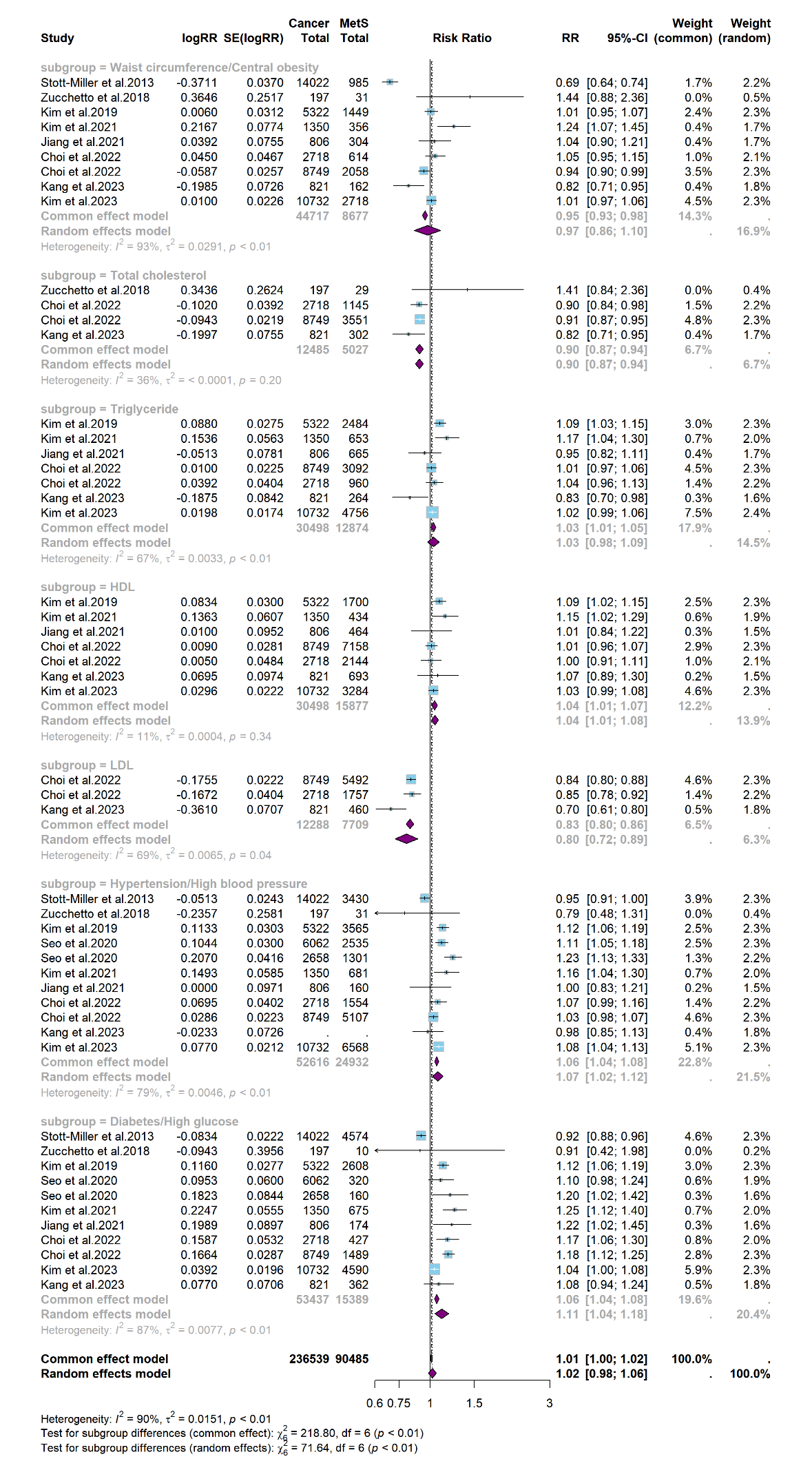


Supplementary Figure 3. Meta-analysis of each component of MetS with risk of Head and Neck Cancer.
